# High copy number variation of cancer-related microRNA genes and frequent amplification of *DICER1* and *DROSHA* in lung cancer

**DOI:** 10.18632/oncotarget.4351

**Published:** 2015-06-22

**Authors:** Karol Czubak, Marzena Anna Lewandowska, Katarzyna Klonowska, Krzysztof Roszkowski, Janusz Kowalewski, Marek Figlerowicz, Piotr Kozlowski

**Affiliations:** ^1^ European Centre for Bioinformatics and Genomics, Institute of Bioorganic Chemistry, Polish Academy of Sciences, Poznan, Poland; ^2^ Molecular Oncology and Genetics Department, Innovative Medical Forum, The Franciszek Lukaszczyk Oncology Center, Bydgoszcz, Poland; ^3^ Department of Thoracic Surgery and Tumors, Nicolaus Copernicus University, Torun, Collegium Medicum, Bydgoszcz, Poland; ^4^ Department of Radiotherapy, The Franciszek Lukaszczyk Oncology Center, Bydgoszcz, Poland; ^5^ Department of Oncology, Radiotherapy and Gynecologic Oncology, Nicolaus Copernicus University, Torun, Collegium Medicum, Bydgoszcz, Poland; ^6^ Department of Thoracic Surgery and Tumors, The Franciszek Lukaszczyk Oncology Center, Bydgoszcz, Poland

**Keywords:** microRNA, DROSHA, DICER1, non-small cell lung cancer NSCLC, MLPA

## Abstract

A growing body of evidence indicates that miRNAs may be a class of genetic elements that can either drive or suppress oncogenesis. In this study we analyzed the somatic copy number variation of 14 miRNA genes frequently found to be either over- or underexpressed in lung cancer, as well as two miRNA biogenesis genes, *DICER1* and *DROSHA*, in non-small-cell lung cancer (NSCLC). Our analysis showed that most analyzed miRNA genes undergo substantial copy number alteration in lung cancer. The most frequently amplified miRNA genes include the following: *miR-30d*, *miR-21*, *miR-17* and *miR-155*. We also showed that both *DICER1* and *DROSHA* are frequently amplified in NSCLC. The copy number variation of *DICER1* and *DROSHA* correlates well with their expression and survival of NSCLC and other cancer patients. The increased expression of *DROSHA* and *DICER1* decreases and increases the survival, respectively. In conclusion, our results show that copy number variation may be an important mechanism of upregulation/downregulation of miRNAs in cancer and suggest an oncogenic role for *DROSHA*.

## INTRODUCTION

Cancer initiation and development are associated with the accumulation of numerous genetic alterations in the cancer genome. These alterations include both small-size mutations and large-scale genomic alterations consisting of copy number variants (CNVs - deletions, duplications or amplifications), as well as copy-number-neutral genomic rearrangements (inversions or translocations). Interactions between these alterations (in certain situations, in addition to germline mutations) allow cancer to clonally evolve due to deactivation of tumor suppressor genes (loss-of-function mutations) and activation of oncogenes (gain-of-function mutations).

Lung cancer is the leading cause of cancer-related death (http://www.who.int/mediacentre/factsheets/fs297/en/; [1]). There are several subtypes of lung cancer, the most common of which is non-small-cell lung cancer (NSCLC). NSCLC can be further divided into adenocarcinoma, squamous-cell carcinoma, and large-cell carcinoma. Lung cancer occurs predominantly in smokers (>60%). Regardless of histological and risk-factor divisions, lung cancer is one of the most genomically heterogeneous type of cancer. Recently, several whole-genome sequencing projects utilizing next-generation sequencing technologies revealed the presence of thousands of small-size mutations in the individual lung cancer genome [2–5], with an almost 10 times higher frequency of mutations in smoker than in non-smoker samples [6]. An even higher level of variation seems to be attributed to copy number alterations. It was shown with the use of SNP-array-based analysis that approximately 50% of the lung cancer genome undergoes recurrent copy number alterations [7]. On average, over 40% of the genome undergoes copy number alteration in individual lung cancers [8]. However, only a small fraction of alterations occurring in cancer genomes are functional (“driver”) mutations; others are “passenger” mutations that occur as a consequence of the general cancer genome destabilization. Although “passenger” mutations are not critical for cancer genome evolution, they are often selected in parallel with closely located or commonly regulated targets of “driver” mutations. The role of “passenger” mutations for particular cancers is mostly unknown (it is not necessarily neutral).

A substantial progress in lung cancer treatment (especially adenocarcinomas) has been made recently due to personalized therapy based on genomic biomarkers. The distinctive biomarkers in lung cancer are mutations in the *epidermal growth factor receptor* (*EGFR*) [9] or gain-of-function translocations and inversions involving the *anaplastic lymphoma receptor tyrosine kinase* (*ALK*) [10]. However, the general prognosis of lung cancer is still poor and its 5-year survival is one of the lowest among cancer patients at approximately 10%. Therefore, many lung cancer studies are currently focused on understanding the impact of genetic alterations on cancer biology and development and on the identification of new prognostic biomarkers.

Among the most intensively studied candidate biomarkers are microRNAs (miRNAs), a class of short (∼21 nt long), single-stranded, noncoding RNAs. MiRNAs are primarily involved in the post-transcriptional regulation of gene expression, either by mRNA degradation or inhibition of translation efficiency [11, 12]. Mature miRNAs are generated in two subsequent steps from long primary precursors (pri-miRNAs). Pri-miRNAs are encoded either by independent transcriptional units or by protein-coding genes. In the first step of miRNA biogenesis that takes place in the nucleus, the secondary precursor (∼60 nt long pre-miRNA), which adopts a hairpin structure, is cleaved out from pri-miRNA by the nuclease DROSHA. Upon export to the cytoplasm, the pre-miRNA is further processed into a miRNA-duplex by the nuclease DICER. One of the miRNA-duplex strands is released, and the other becomes the mature miRNA that, as a key element of the miRNA-induced silencing complex (miRISC) recognizes complementary target sequences usually located within the 3′ untranslated regions of mRNAs.

The biological functions of most miRNAs identified so far (miRBase; http://www.mirbase.org; [13, 14] remain unknown. However, it has been well documented that miRNAs downregulate numerous genes and either stimulate or inhibit many important biological processes and diseases, including cell proliferation and differentiation, apoptosis, development and cancer [15–18].

The role of miRNAs in the development of cancer was first identified in chronic lymphocytic leukemia in 2002 [19]. Since then, it has been shown that overexpression or downregulation of certain miRNAs contributes to the development, progression and metastasis of many types of cancer. Such miRNAs can therefore be classified as either oncogenes (oncomirs) or tumor suppressors [20]. It has also been shown that some miRNAs, such as *miR-21*, *miR-205* or *miR-155*, seem to be universal for different cancers [12].

There have been numerous studies of miRNA expression in lung cancer, and many miRNAs that are specifically over- or underexpressed in lung cancer or in particular lung cancer subtypes were identified. For example, it was shown that 6 miRNAs constituting the polycistronic miRNA cluster, *miR-17/92*, are overexpressed in lung cancer and enhance cell proliferation [21]. It was later shown that an elevated level of these miRNAs may be detected in the plasma of lung cancer patients [22, 23] and is associated with poor disease prognosis [24]. Other miRNAs consistently found to be either overexpressed or underexpressed in lung cancer are *miR-21*, *miR-210* and *miR-126*. However, it should be noted that substantial discordances between miRNA profiling results also exist.

Although the functional relevance of some of the miRNAs that are differentially expressed in lung cancer has been demonstrated (e.g., [25–27]), the roles of most of these miRNAs in cancer are unknown or poorly recognized. One factor that may shed more light on the role of particular miRNAs in cancer is the mechanism underlying their aberrant expression. Among the most pronounced mechanisms underlying aberrant expression in cancer are point mutations, epigenetic modifications and copy number alterations. However, it has been suggested that point mutations and epigenetic modifications are not important factors in the global miRNA regulation in lung cancer [24, 28]. It has also been shown that miRNA genes are overrepresented and cluster in genomically fragile sites and other regions that undergo frequent copy number changes in cancer genomes. Thus, it has been suggested that somatic copy number variation may lead to the activation/deactivation of miRNAs in cancer [29, 30]. For example, systematic analysis of three cancer types (ovarian, breast, and melanoma) with the use of comparative genome hybridization microarrays showed that 37% (ovarian) to 89% (melanoma) of analyzed miRNA genes undergo copy number changes [30]. There are known examples of both miRNA- and protein-coding genes whose expression in cancer is either increased or decreased by their copy number variation. These high-copy-number amplifications and recurrent deletions (loss of heterozygosity) are often used as a confirmation of oncogenic and tumor-suppressive function of the analyzed gene, respectively. The role of copy number variations in the regulation of miRNAs in cancer and the potential cancer-related implications have been reviewed before [31–33]. The most recent review provides an excellent summary and discusses this notion using ovarian cancer as an example [33].

In this study, with the use of homemade multiplex ligation-dependent probe amplification (MLPA) assays, we analyzed the somatic copy number variation of 14 miRNA genes consistently found to be either over or underexpressed in lung cancer. Additionally, we analyzed the copy number variation of *DICER1* and *DROSHA*, two main miRNA biogenesis genes. We analyzed these genes in 254 NSCLC samples and observed high copy number variation in most of the analyzed genes. Among the frequently amplified miRNA genes were *miR-21*, *miR-17/92* and *miR-155*, which are commonly recognized as oncomirs, as well as *miR-30a* and *miR-30d* which were downregulated in lung cancer. Surprisingly, a high average copy number value and frequent amplifications were present in both miRNA biogenesis genes. We also showed that amplification of *DROSHA* is not driven by other closely located oncogenes. The most frequently deleted miRNA gene turned out to be *miR-126*, which is commonly found to be downregulated in lung cancer. Our analysis showed that a substantial fraction of differentially expressed miRNAs may be explained by and are consistent with the copy number variation of their genes.

## RESULTS

### Selection of miRNA genes for copy number analysis in lung cancer

**Figure 1:**
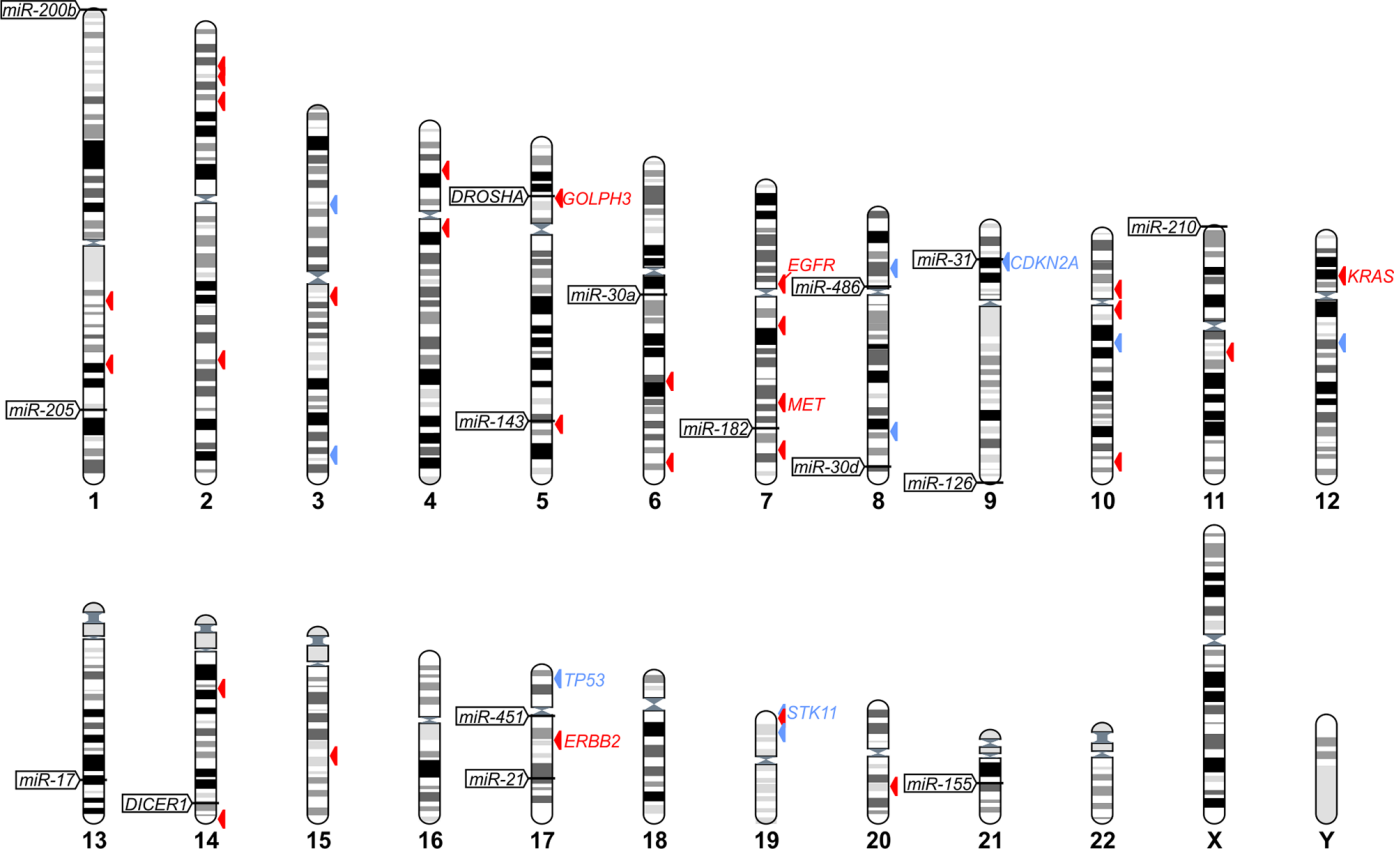
The positions of selected miRNA and miRNA biogenesis genes in human genome The positions of miRNA and miRNA biogenesis genes are indicated on chromosome ideograms (left-hand side). Arrowheads on the right-hand side of the ideograms indicate lung cancer relevant genes catalogued in COSMIC: the Cancer Gene Census (associated with one of the following terms: “lung”, “NSCLC” or “multiple tumor types”), the most reliable list of cancer-related genes annotated and curated by the Wellcome Trust Sanger Institute (originally published in [78]). Additionally, the position of *GOLPH3*, which is discussed in this study, is indicated. Red and blue arrowheads indicate oncogenes and tumor suppressor genes, respectively. IDs of the most relevant genes are indicated next to the arrowheads. The figure was prepared with the use of the “Ensembl karyotypes” tool available on the Ensembl portal.

To select miRNA genes for our analysis, we took advantage of two recently published meta-analysis studies [34, 35] summarizing the results of dozens of whole-genome miRNA expression studies in lung cancer (references within [34, 35]). Although these two studies utilized completely different strategies of meta-analyses, the top significantly up- and downregulated miRNAs identified in both studies overlap perfectly (with minor differences in the order of identified miRNAs). Based on these meta-analyses, we selected 6 genes/genomic regions (*miR-21*, *miR-210*, *miR-182*, *mir-31*, *mir-200b*, *mir-205*) encoding miRNAs most consistently identified as upregulated, and 6 genes (*miR-126*, *miR-30a*, *miR-30d*, *miR-486*, *miR-451a*, *miR-143*) encoding miRNAs most consistently identified as downregulated in lung cancer. Additionally, for our analysis we selected the genomic regions of *miR-155* and *miR-17* (identified in one meta-analysis), which were consistently associated with poor prognosis of lung cancer, as well as two genes (*DICER1* and *DROSHA*) encoding miRNA processing enzymes. The genomic positions of all selected genes are indicated in Figure 1, and the criteria for their selection are summarized in Table 1. Note that some of the selected miRNA genes encompass miRNA clusters (e.g., *miR-17/92* and *miR-143/145*).

**Table 1:** List of miRNA and miRNA biogenesis genes selected for analysis

			expression change top-ranked in meta-analysis		other lung cancer relevant features		
analyzed loci IDs		miRs in cluster	Vosa et al.^[35]^ [corrected *p*-value]	Guan et al.^[34]^ [mean fold change]	association with poor prognosis	potential biomarkers^B^	frequently deregulated in other solid tumors
miRNA and miRNA-cluster genes	*miR-21*		↑ 2E-14	↑ 4.4	+ ^[24, 80, 81]^	+ ^[23, 82, 83]^	+ ^[12, 84]^
	*miR-210*		↑ 6E-11	↑ 2.7		+ ^[23]^	
	*miR-182*	*182*^E^, *183*^E^, *96*	↑ 3E-8, 4E-2	↑ 6.3, 5.9		+ ^[23, 83, 85, 86]^	+ ^[84]^
	*miR-31*		↑ 1E-4	↑ 2.89			+ ^[84]^
	*miR-200b*	*200b*^E^, *200a*, *429*	↑ 1E-3	↑ 3.7		+ ^[82, 83]^	+ ^[84]^
	*miR-205*		↑ 7E-3	↑ 23.2		+ ^[83]^	
	*miR-126*		↓ 7E-12	↓ .33		+ ^[23, 86]^	+ ^[56]^
	*miR-30a*	*30a*^E^, *30b*	↓ 1E-9	↓ .36			
	*miR-30d*		↓ 2E-8	↓ .34			
	*miR-486*		↓ 4E-7	↓ .39		+ ^[23, 82]^	
	*miR-451a*	*451a*^E^, *4732*, *144*	↓ 7E-5	↓ .37		+ ^[83]^	
	*miR-143*	*143*^E^, *145*^E^	↓ 7E-4, 1E-3	↓ .33, .23	+ ^[24]^	+ ^[83]^	
	*miR-155*		↑* ^[12, 24, 80, 85]^		+ ^[24, 81]^	+ ^[85]^	+ ^[12, 84]^
	*miR-17*	*17*, *18a*, *19a*, *20a*, *19b-1*, *92a-1*	↑* ^[12, 51, 80]^		+ ^[24]^		+ ^[12, 84]^
miRNA biogenesis genes	*DICER1*		↑/↓* ^[87–89]^		+ ^[87, 88]^		+ ^[90–93]^
	*DROSHA*		↑* ^[87]^		+ ^[87]^		+ ^[73, 91–93]^

E miRNAs reported as top-ranked in both meta-analyses;

* expression changes non top-ranked or not analyzed in meta-analyses;

B altered in plasma/serum/blood/sputum of lung cancer patients and/or associated with early stage NSCLC ^[23, 82, 85, 86]^

### MLPA assays design

To analyze the somatic copy number variation of selected genomic regions, we designed two MLPA assays, each covering 7 miRNA genes and 1 miRNA biogenesis gene. Each miRNA or miRNA cluster region was covered by two MLPA probes located in close proximity (mostly within 1 kb) to an annotated pre-miRNA sequence, preferentially on both sides of the pre-miRNA sequence. Each of the miRNA biogenesis genes (*DICER1* and *DROSHA*) was covered by 3 MLPA probes located in exons distributed about equally across the genes. Additionally, each MLPA probe-set contained 4 control probes specific for different chromosomes. The exact genomic location and sequence of each probe is indicated in Supplementary Table S1. MLPA assays were designed and generated according to a strategy developed and have been described in detail previously [36, 37]. We validated the performance of the assays with the panel of reference non-cancer DNA samples and showed that all covered genomic regions are genetically stable and always occur in 2 copies.

### Analysis of the somatic copy number variation of selected miRNA genes

**Figure 2:**
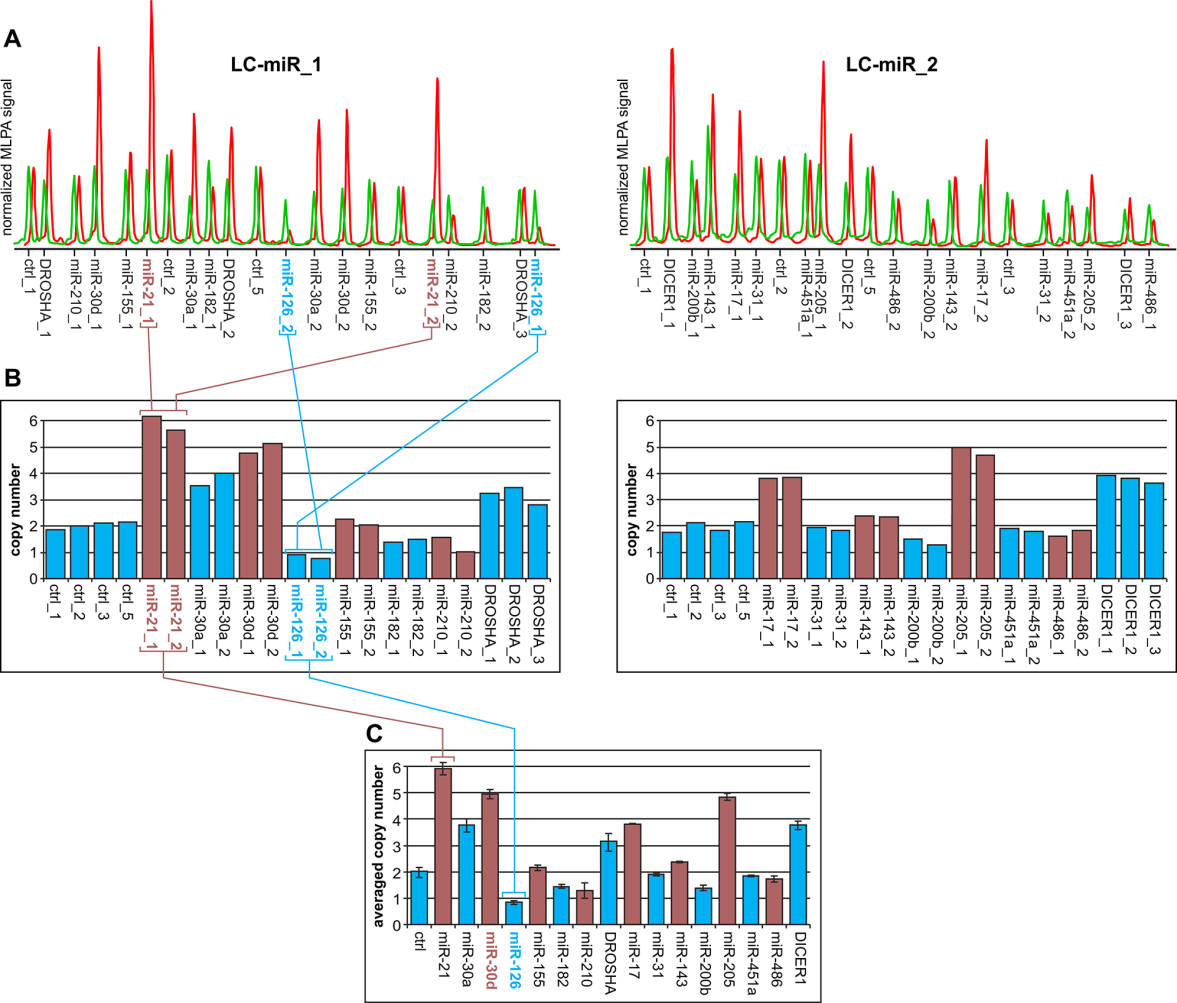
Copy number analysis of the selected genomic regions in a representative lung cancer sample **A.** Electropherograms of MLPA results obtained with the use of LC-miR_1 (left-hand) and LC-miR_2 (right-hand) MLPA assays. The electropherograms of the cancer sample (red) are presented along the electropherograms from a reference non-cancer sample (green) and normalized against the signal of control probes. Probe IDs are indicated below the electropherograms. The probe signals (peak heights) correspond to the copy number of targeted regions. **B.** Bar plots (corresponding to the electropherograms of the cancer sample shown above (A)) represent the copy number value (y-axis) of each probe (x-axis) normalized by comparison of its signal in cancer samples to the corresponding signal in reference sample. The colors were used purely for sake of visualization purposes to better distinguish probes of subsequent genomic regions. Note that the signals of probes specific to the same genomic region are synchronized (e.g., probes miR-21_1 and miR-21_1 or miR-126_1 and miR-126_2; indicated in panels A and B). **C.** Bar plot representing the average copy number values of investigated regions in analyzed samples. Whiskers indicate maximum and minimum copy number values detected in particular regions, as shown in panel B. Note that genomic regions in which the difference between the maximum and minimum signal was higher than one-third of an average copy number value were excluded from further analysis (*miR-210*).

**Figure 3:**
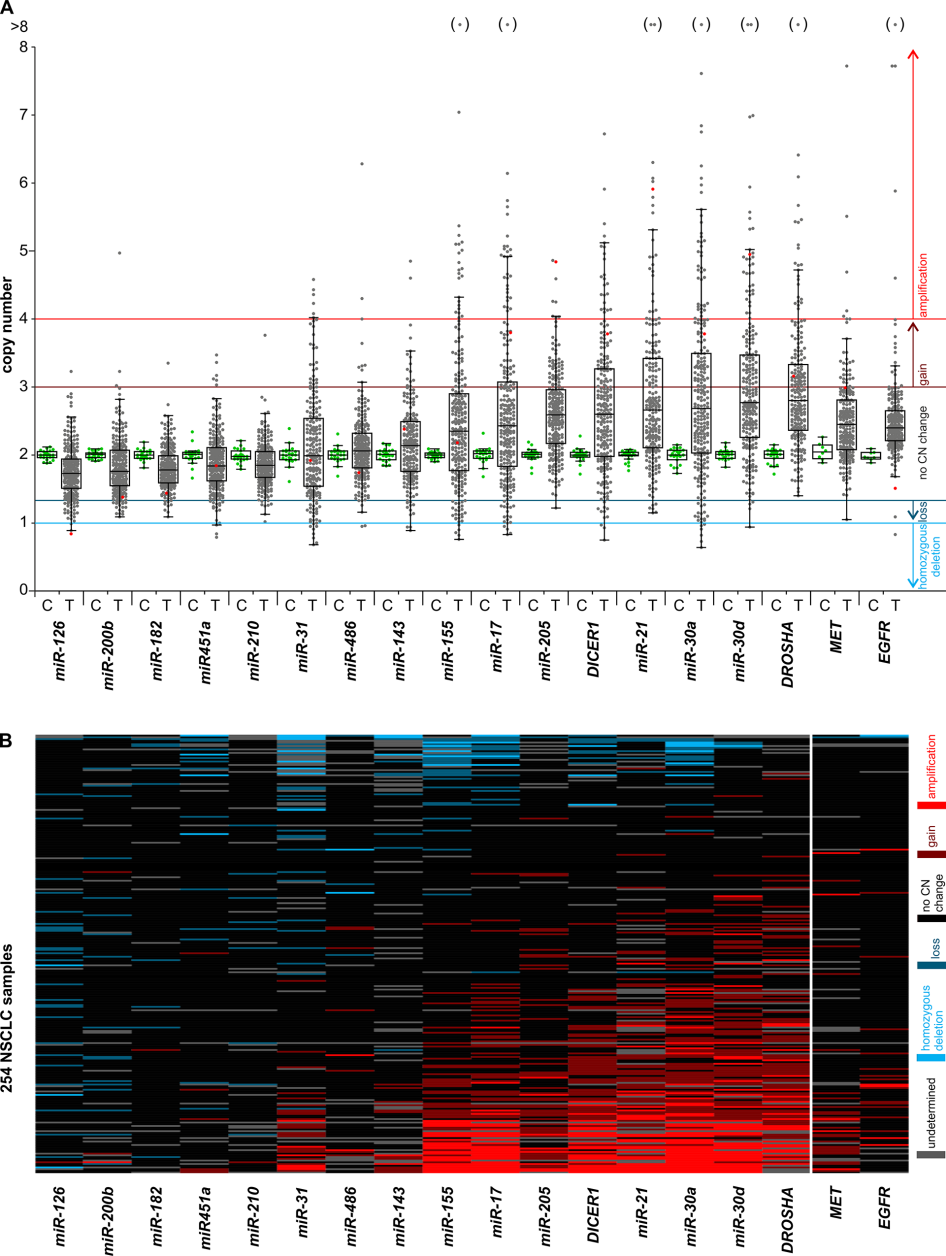
Graphical summary of the copy number variation of the analyzed genes in NSCLC samples The graph shows the results of copy number analysis of the selected miRNA and miRNA biogenesis genes as well as two lung cancer related oncogenes, *MET* and *EGFR*. **A.** The graph shows the relative copy number values (y-axis) of selected genes (x-axis) of all studied samples. The genes were ordered from the lowest to highest median copy number value. Each dot represents the copy number value of individual control (C – green dot) or lung cancer (T – grey dot) samples. Red dots indicate copy number values of the representative lung cancer sample, analysis of which is shown in Figure 2. Dots in brackets (above) indicate samples with a copy number value >8. Color lines represent threshold values of homozygous deletions, losses, gains and amplifications. The outlined Tukey box-and-whisker plots indicate 1st quartile, median and 3rd quartile, and summarize the distribution of the presented copy number values. **B.** The heatmap graph showing the distribution of copy number categories of analyzed genes (columns) in 254 lung cancer samples (rows). The genes (from the left) and samples (from the top) were ordered from the lowest to highest average copy number value. Copy number categories are indicated by colors as shown in the legend on the right.

With the use of the developed MLPA assay, we analyzed 254 NSCLC samples and determined the relative copy number value of all analyzed regions in these samples. As shown in Figure 2, the signals of probes representing particular regions in most cases are strongly synchronized. If one probe in a particular region indicates a copy number increase, the other probe or probes in these regions also show similar levels of copy number increase. As each MLPA probe recognizes different target sequence, such a correlation provides independent validation of the obtained results. The copy number value of a particular region was calculated as the average of the copy number values of the respective probes. The regions for which inter-probe variation was too high were considered uninterpretable and were excluded from further analysis. The relative copy number values of all analyzed regions are shown in Supplementary Table S2 and graphically summarized in Figure 3. As analyzed NSCLC samples are contaminated with different amounts of normal DNA (in most samples percentage of tumor cells (PTC) is >50%, and an average PTC is approximately 70%) the estimated copy number changes are generally diluted and lower than in actual cancer cells. For comparison, copy number values corrected for PTC (dilution) factor are shown in Supplementary Figure S1. As shown in Figure 3 and Supplementary Figure S1, the average copy number of analyzed regions differs substantially and is highest for *DROSHA*, *miR-30d*, *miR-30a*, *miR-21*, *DICER1*, *miR-205*, *miR-17*, and *miR-155* and lowest for the *miR-126* region (Table 2).

**Table 2:** Summary of copy number changes observed in analyzed miRNA and miRNA biogenesis genes in NSCLC samples

	expression	copy number: median (average)	gains: number (%)	amplifications: number (%)	losses:number (%)	hom. deletions: number (%)	informative samples #
***miR-126***	↓	1.73 (1.76)	1 (0.4)	0 (0)	26 (10.8)	3 (1.2)	241
***miR-200b***	↑	1.76 (1.84)	2 (0.9)	1 (0.4)	18 (7.7)	0 (0)	235
***miR-182***	↑	1.78 (1.81)	1 (0.4)	0 (0)	9 (3.8)	0 (0)	240
***miR-451a***	↓	1.84 (1.89)	6 (2.5)	0 (0)	12 (5.0)	4 (1.7)	239
***miR-210***	↑	1.85 (1.87)	1 (0.4)	0 (0)	9 (4.0)	0 (0)	227
***miR-31***	↑	1.99 (2.11)	22 (10.0)	6 (2.7)	20 (9.1)	8 (3.7)	219
***miR-486***	↓	2.06 (2.11)	7 (3.0)	2 (0.9)	3 (1.3)	2 (0.9)	233
***miR-143***	↓	2.14 (2.16)	12 (5.9)	2 (1.0)	8 (3.9)	3 (1.5)	205
***miR-155***	↑	2.33 (2.50)	33 (13.5)	23 (9.4)	21 (8.6)	5 (2.0)	245
***miR-17***	↑	2.42 (2.62)	39 (16.0)	28 (11.5)	15 (6.1)	5 (2.0)	244
***miR-205***	↑	2.59 (2.61)	45 (19.0)	8 (3.4)	1 (0.4)	0 (0)	237
***DICER1***	↓/↑	2.60 (2.69)	51 (21.8)	23 (9.8)	8 (3.4)	3 (1.3)	234
***miR-21***	↑	2.63 (2.90)	48 (22.7)	25 (11.8)	6 (2.8)	0 (0)	211
***miR-30a***	↓	2.67 (2.90)	59 (23.8)	40 (16.1)	14 (5.6)	6 (2.4)	248
***miR-30d***	↓	2.77 (3.02)	63 (26.6)	35 (14.8)	3 (1.3)	1 (0.4)	237
***DROSHA***	↑	2.79 (3.00)	67 (30.7)	23 (10.6)	0 (0)	0 (0)	218
***MET***	↑	2.45 (2.50)	23 (9.9)	5 (2.2)	1 (0.4)	0 (0)	232
***EGFR***	↑	2.41 (2.55)	13 (5.3)	5 (2.0)	1 (0.4)	1 (0.4)	246

Pronounced copy number changes may be more indicative of the role of a particular region in cancer. Therefore, based on criteria similar to those applied before [38, 39], we classified the identified copy number changes to the following categories (from highest to lowest copy number): amplifications (≥4 copies; ≥2x increase), gains (≥3 copies; ≥1.5x increase), losses (≤1.33 copies; ≤1.5x decrease) and homozygous deletions (≤1 copy; ≤2x decrease). The categorized copy number changes of all analyzed samples are visualized in a heatmap graph (Figure 3B) and are summarized in Table 2. The results indicate that the number of amplifications detected in particular genes generally correlates with an increase in the average copy number value of these genes. The highest frequency of amplifications was observed in *miR-30a*, *miR-30d*, *miR-21*, *miR-17*, *DROSHA*, *DICER1*, and *miR-155*. In some of these genes both amplifications and isolated cases of deletions were detected. The genes for *miR-182*, *miR-200b* and *miR-210* turned out to be relatively stable, showing neither amplifications nor homozygous deletions. Only a few homozygous deletions but no amplifications were detected in *miR-126* and *miR-451a*. For comparison, the results of copy number changes of genes analyzed in this study are presented along with corresponding results of two oncogenes, *EGFR* and *MET*, obtained previously with the use of a similar methodology [40].

### Extended analysis of the *DROSHA* locus on chromosome 5

**Figure 4:**
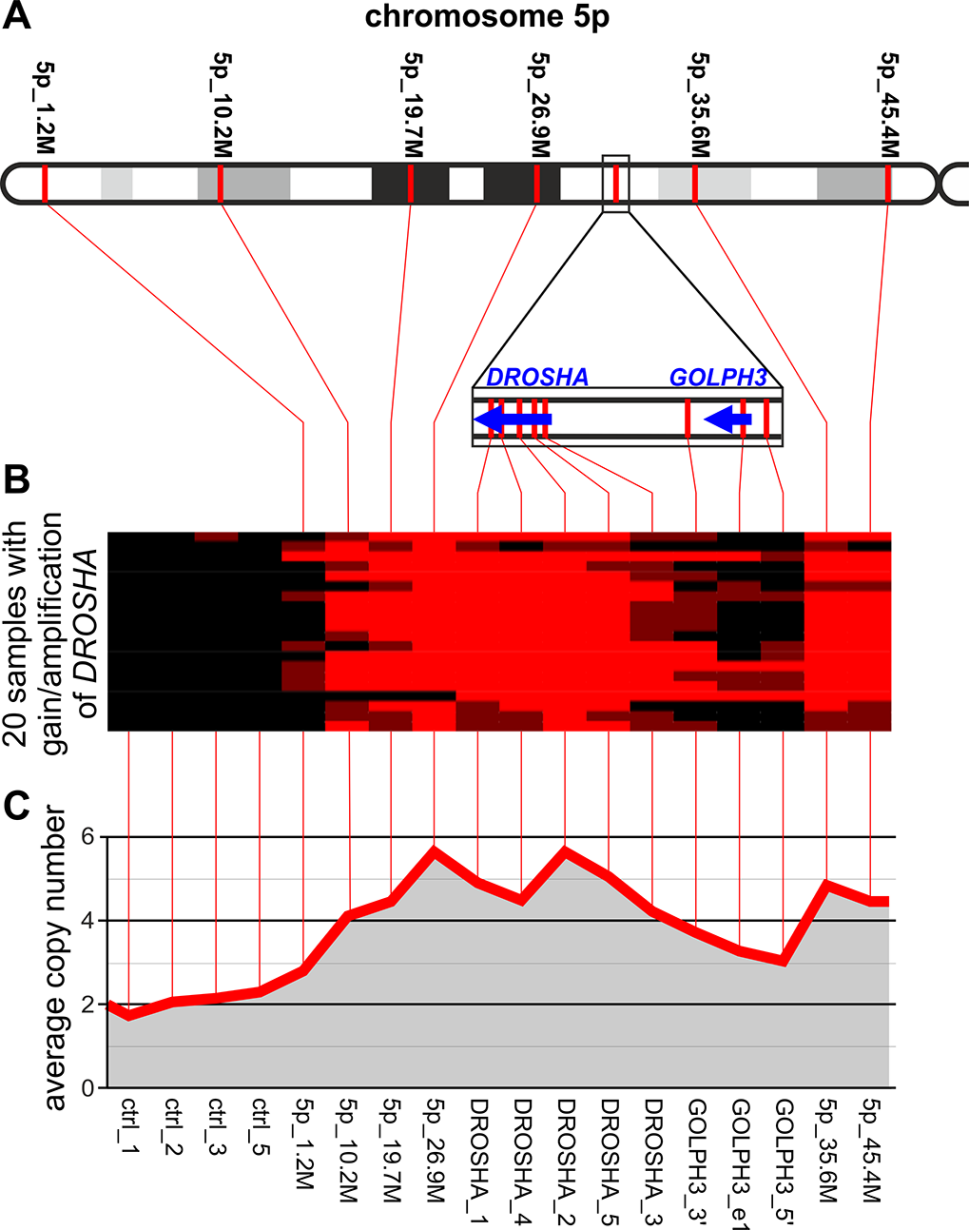
Analysis of copy number changes in the 5p-arm in NSCLC samples with gain/amplification of *DROSHA* **A.** The schematic map of the 5p-arm with indicated positions of LC-5p MLPA probes (spaced by approximately 10 Mbp). The *DROSHA*/*GOLPH3* region, more densely covered by MLPA probes, is zoomed in on below. **B.** A heatmap graph showing copy number categories of all analyzed samples (18 with *DROSHA* amplification and 2 with *DROSHA* gain; rows) in control regions and in regions along the 5p-arm (columns). Red, brown and black colors indicate amplification, gain and no copy number change, respectively. **C.** A line-graph indicating the average copy number values (y-axis) of analyzed samples in control regions and in regions along the 5p-arm (x-axis). Note that in B and C, the spacing of consecutive probe signals depicted on the graphs does not correspond to their exact genomic distance.

One of the genes with the highest average copy number and the highest frequency of amplifications was *DROSHA*. To investigate whether copy number increases in *DROSHA* result from amplification of other nearby genes or regions, we designed an additional MLPA assay (LC-5p) covering the short arm of chromosome 5 (5p-arm). Except for the 4 control probes that were used before, the assay was composed of (i) 6 probes more or less evenly distributed along the entire chromosome arm, (ii) 5 probes covering the *DROSHA* gene (3 probes used before and 2 new probes), and 3 probes covering the *GOLPH3* gene, recently identified as oncogene [41] located in close proximity (∼0.5 Mb upstream) to *DROSHA*. The locations of the probes are indicated in Figure 4A and Supplementary Table S1. With the use of the developed assay, we analyzed 20 samples selected based on the increased signal of *DROSHA* observed in the first experiment (18 amplifications and 2 gains). The copy number values of *DROSHA* determined by two independent experiments (with the use of LC-miR_1 and LC-5p assays) showed a very strong correlation (*R* = 0.92, *p* < 0.0001, data not shown). As shown in Figure 4, increased copy number is observed along almost the entire 5p-arm and no specific region shows sign of focal amplification. The region of amplifications observed in particular samples extends from the probe 5p_10, 2M to the probes covering *DROSHA*, and usually does not encompass *GOLPH3* (Figure 4). The above experiment clearly demonstrates that amplification of *DROSHA* is part of a chromosome-level amplification of the 5p-arm and is not a “passenger” effect of focal amplification of some other oncogene.

### Survival analysis of patients stratified by copy number categories of miRNA and miRNA biogenesis genes

**Figure 5:**
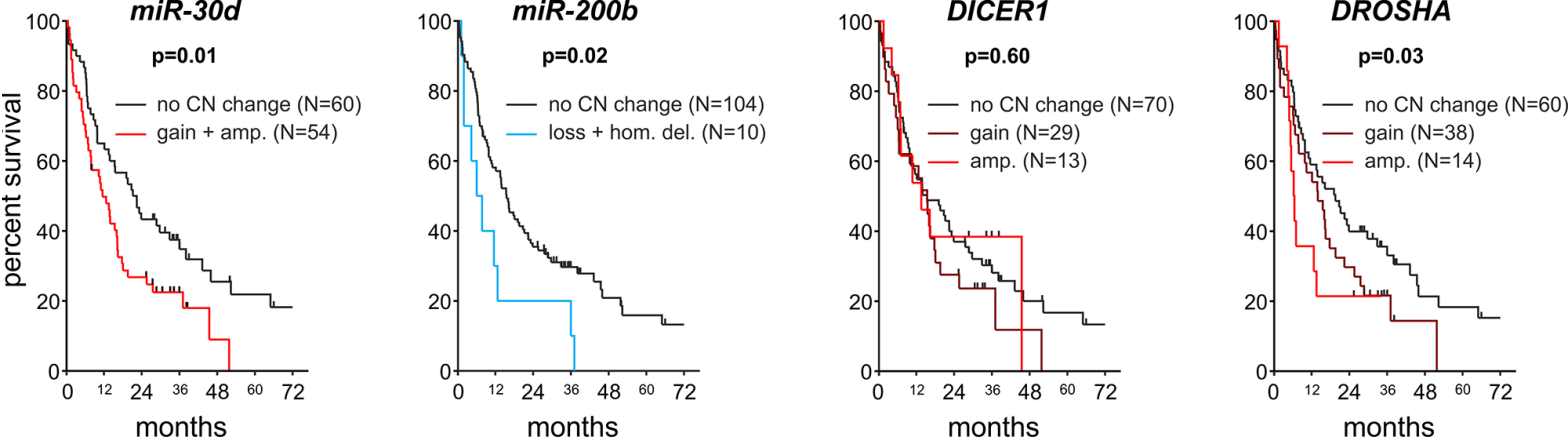
Survival analysis of NSCLC patients Kaplan-Meier graphs presents the survival of patients stratified based on copy number categories of (from the left) *miR-30d*, *miR-200b*, *DICER1* and *DROSHA*.

The overall survival data were available for 120 of the analyzed patient samples. Median overall survival of these patients was 416 days (14 months). Kaplan-Meier survival analysis of patients grouped based on copy number categories showed significant decreases in the survival of patients with the *miR-200b* deletion (log-rank test, *p* = 0.022) and patients with gain or amplification of *miR-30d* (*p* = 0.013) (Figure 5). This corresponds to a lower 5-year survival rate (0%) of patients with the above mentioned copy number aberrations compared to patients without the aberrations in *miR-200b* (6%) and *miR-30d* (10%).

Similar analyses performed for *DICER1* and *DROSHA* showed that samples with an increased copy number of *DROSHA* have significantly decreased survival and that the survival rate corresponds to the degree of copy number increase (log-rank test for trend, *p* = 0.032) (Figure 5).

### Association of clinical data with copy number categories of miRNA and miRNA biogenesis genes

The copy number categories of any of the analyzed regions showed substantial association with the sex or age of the analyzed patients (Supplementary Table S3). Somewhat higher average age of diagnosis showed samples with *miR-126* deletion (with del/without del; 64.9/61.2 years; *p* = 0.046), *miR-451a* deletion (with del/without del; 66.8/61.2 years; *p* = 0.041), and with *miR-31* deletion (with del/without del; 65.7/61.0 years; *p* = 0.017). It has to be noted, however, that these associations are only marginally significant on the nominal level but not after adjustment for multiple comparisons. We also did not find any significant association of copy number categories with clinical data, such as stage of lung cancer at time of sample collection and metastasis/progression/remission status during the last examination (Supplementary Table S3). It has to be noted, however, that clinical data were available only for part of the analyzed samples (*N* = 120) and therefore, the lack of association may result from relatively low statistical power of our analysis.

### Computational analysis of the association of *DICER1* and *DROSHA* copy number categories with their expression and cancer patient survival

**Figure 6:**
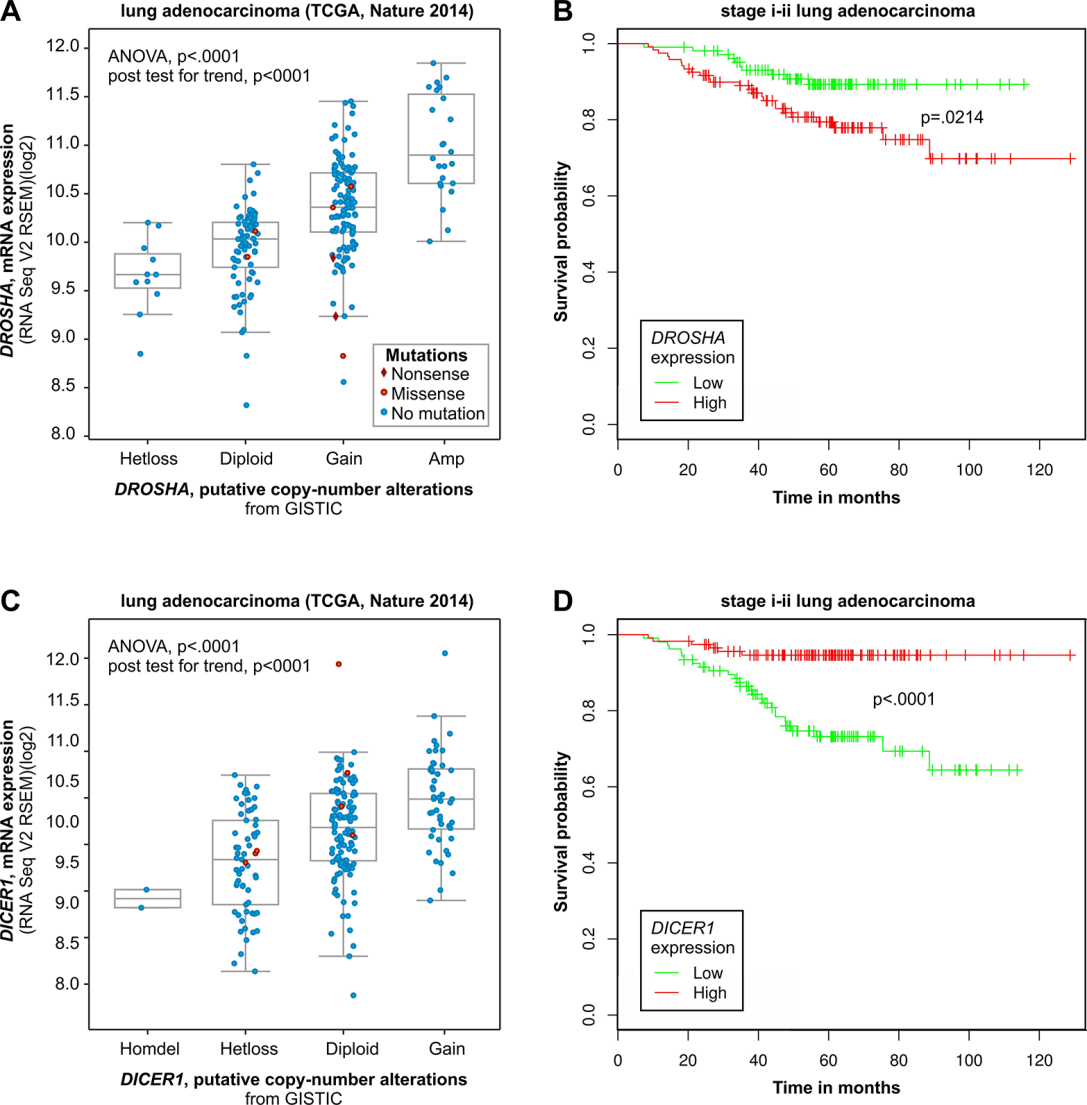
Computational analysis of clinical (survival) and oncogenomic data of *DROSHA* and *DICER1* Mutual relation between copy number and expression (oncogenomic data) of *DROSHA* **A and B.** and *DICER1* **C and D.** and the relation of their expression to survival of cancer patients. A) and C) Correlation analysis of copy number categories and expression level performed with the use of a dataset (lung adenocarcinoma TCGA [79]) deposited and tools available in cBioPortal for Cancer Genomics. B) and D) Survival analysis performed with the use of a dataset (stage i-ii lung adenocarcinoma; GEO: GSE31210) deposited in and tools available from the PPISURV web portal.

Because we do not have access to mRNA/cDNA material or the expression data for our samples to determine whether copy number changes in *DICER1* and *DROSHA* correlate with their expression, we used data deposited in the cBioPortal for Cancer Genomics [42, 43]. As shown in Figure 6, there is a dose-dependent correlation between the copy number categories and the expression of *DICER1* and *DROSHA* in lung cancer (based on TCGA Cancer Genome ATLAS data [44]). A similar correlation can be observed in other cancers analyzed in different studies (Supplementary Figure S2). Further analysis with the use of another oncogenomic tool, PPISURV [45], showed that the increased expression of *DROSHA* generally (across cancers/datasets) correlates with decreased survival (Figure 6B and Supplementary Figure S2). In most deposited datasets/cancer types, including lung cancer, correlations show the same negative direction (in 6 of 36 datasets association show significance at *p* ≤ 0.05). Similar analysis performed for *DICER1* shows the opposite effect of increased expression. In most deposited datasets, increased expression of *DICER1* shows the association (positive correlation) with increased survival (14 of 42 datasets show association at *p*-≤ 0.05; Figure 6B and Supplementary Figure S2).

## DISCUSSION

With the use of two homemade MLPA assays, we analyzed the copy number variation of 14 miRNA genes reported as either over- or underexpressed in lung cancer. Additionally, we analyzed two critical miRNA biogenesis genes, *DROSHA* and *DICER1*. Each analyzed gene was tested by at least two independent MLPA probes, providing additional internal validation for the obtained results. To avoid any potential false results, the substantially discordant signals of matched probes were excluded from analysis. A similar strategy of somatic copy number variation analysis may be applied to almost any genomic region of interest in cancer samples. It should be noted, however, that the obtained copy number values are relative and to some extent may depend on the copy number variation of selected control regions (probes).

The analysis showed a substantial somatic copy number variation (both gains and losses) of all selected regions in cancer samples (compared variation in cancer vs. control, non-cancer samples; Figure 3). However, the observed copy number alterations are not random, and some regions show a substantial increase (frequent amplifications), while the others show decrease in the average copy number value. The genes showing the highest average level of copy number include *miR-30d*, *miR-30a*, *miR-21*, *miR-205*, *miR-17*, *miR-155* as well as *DROSHA* and *DICER1*. Surprisingly, the average copy number and the frequency of amplifications of some of these genes (e.g., *DROSHA, miR-30d, miR-30a* and *miR-21)* are substantially higher than the corresponding values of well-known lung cancer-related oncogenes, *EGFR* and *MET*, analyzed in the same set of samples. In contrast, *miR-126* showed the lowest average copy number and a relatively high frequency of deletions and homozygous deletions. It should be noted, however, that due to the contamination of cancer samples with normal DNA and the inherent lower amplitude of copy number losses than copy number gains, the power of our analysis to detect deletions was substantially lower than the power to detect copy number gains/amplifications. Some genes, such as *miR-31*, show a relatively high frequency of both gains/amplifications and deletions.

As expected, the copy number variation of analyzed miRNAs does not correlate perfectly with the global expression changes of these miRNAs observed in lung cancer. However, our results indicate that copy number gains/amplifications may contribute substantially and may be an important mechanism underlying overexpression of miRNAs such as *miR-21*, *miR-17*, *miR-205* or *miR-155*. In short, these miRNAs are the best known oncomirs implicated not only in lung cancer but also in many other types of cancer (reviewed in [20, 26, 46, 47]). *MiR-21* was originally recognized as an antiapoptotic miRNA [48] that was strongly overexpressed in most types of cancer. Later, it was shown that *miR-21* promotes growth, metastasis and invasiveness, as well as chemo- and radioresistance of NSCLC, most likely by targeting tumor suppressor *PTEN* [49, 50]. In our experiment, *miR-17* represents 6 miRNAs coded in the *miR-17/92* cluster located within intron 3 of the *C13orf25* on chromosome 13. It was shown that the *miR-17/92* cluster may be upregulated by gene amplification, which is consistent with our results, or by *MYC* overexpression. It was also shown that upregulation of the *miR-17/92* cluster promotes cell proliferation and inhibits lung cell differentiation (the role of *miR-17/92* cluster was reviewed in [51]). *MiR-205* acts either as a tumor suppressor or as an oncogene. As an oncogene, it promotes tumor initiation, progression, resistance to therapies and inhibits apoptosis. It was shown that the oncogenic role of *miR-205* is expressed mostly by downregulation of tumor suppressors such as *PTEN* and *SHIP2* (references within [46]). *MiR-155* is encoded by the non-protein-coding gene *BIC*, originally identified as B-cell integration cluster for the avian leukosis virus, inducing lymphomas [52]. It was shown that *miR-155* targets several tumor suppressors such as *SOCS1*, *FOXO3*, and *VHL* and is involved in the regulation of cell survival, growth, chemosensitivity and tumor angiogenesis [53–55].

On the other hand, *miR-126* showed the lowest average copy number and frequent deletions in our study and is also recurrently found as downregulated in lung cancer. *MiR-126* was recognized as a tumor suppressor in most of the cancers studied. It was shown that *miR-126* may negatively control and inhibit cell proliferation, migration, invasion, and cancer cell survival. Among the validated targets of *miR-126* are such oncogenes as *ADAM9*, *CRK*, *EGFL7*, *HOXA9*, *IRS1*, *KRAS*, *PI3K*, *SLC7A5*, *SOX2*, and *VEGF* (reviewed and references within [56]).

The example of miRNAs which show discordant directions of expression and copy number changes are *miR-30a* and *miR-30d*, both belonging to *miR-30* family. *MiR-30a* and *miR-30d* belong to the miRNAs most frequently reported to be downregulated in lung cancer. On the other hand, these two miRNAs exhibit average copy number values and amplification frequencies that are among the highest of the genes analyzed in our study. It should be noted, however, that the copy number increases in *miR-30d* observed in our study correspond well to the results obtained previously by Li et al.. They showed that *miR-30d* is frequently amplified in different types of cancer (∼30%) including lung cancer (27%), and that amplification of *miR-30d* correlates with its overexpression [57]. It was also shown that *miR-30d* downregulates many cancer-related genes, including apoptotic caspase *CASP3*, and is involved in the upregulation of such processes as cell proliferation, apoptosis, and migration [57]. The above facts strongly suggest the oncogenic character of *miR-30d*. Additionally, our results suggest that increased copy number of *miR-30d* (gains or amplifications vs. others) correlate with significantly reduced survival (Figure 5). On the other hand, *miR-30a* has been frequently implicated as a tumor suppressor. It was shown that *miR-30a* targets and downregulates the transcription factor Snai1 and consequently inhibits the epithelial-to-mesenchymal transition (EMT), invasion, mobility and metastasis of NSCLC cells [25]. The opposite characteristics of these two miRNAs may be reflected by the different frequency of deletions of these two miRNAs observed in our study. Although *miR-30a* showed a substantially increased average copy number, it was also one of the most frequently deleted in our analysis. Of our analyzed samples, 20 (8%) showed deletion of *miR-30a*, including 6 samples (2.4%) with homozygous deletions. For comparison, only 5 samples showed deletion of *miR-30d*.

Another example of miRNA with opposite trends in global expression and copy number changes is *miR-200b*. Although upregulation of *miR-200b* was recurrently identified in lung cancer, its character suggests it is likely a tumor suppressor. *MiR-200b* belongs to the *miR-200* family that maintains the general characteristics of the epithelia and inhibits EMT, tumor cell motility, and invasiveness ([58] and references within). Among the experimentally identified and validated targets of *miR-200b* are numerous genes involved in the regulation of cytoskeletal organization and cell morphology in addition to *EGFR* [58]. Additionally, our analysis showed significantly decreased survival of patients with either deletion or homozygous deletion of *miR-200b*.

In addition to miRNA genes, we analyzed also two key miRNA biogenesis genes, *DICER1* and *DROSHA*. Both of these genes, but especially *DROSHA*, show substantial copy number increases and frequent high-copy number amplifications in analyzed samples. Review of the Cancer Gene Census (COSMIC database) reveals no proto-oncogene in close proximity of either *DROSHA* or *DICER1* that might drive their amplification. However, meticulous review of the literature allowed us to identify *GOLPH3* located in direct proximity (∼600 kb upstream) of *DROSHA*. *GOLPH3* encodes a Golgi-localizing protein that was recently identified as a candidate oncogene driving the amplification of the 5p13 region. This amplification has frequently been observed in multiple solid tumors, including lung cancer [41]. It was shown that Golph3 enlarges cell size, enhances growth-factor-induced mTOR signaling in human cancer cells, and increases the sensitivity to an mTOR inhibitor [41]. The detailed analysis showed that the region of amplification comprising *GOLPH3* is very narrow and does not extend to *DROSHA*. However, the frequency of *GOLPH3* amplification in lung cancer observed previously (56%) corresponded well to the frequency of gains/amplifications of *DROSHA* observed in our study (42%). To verify whether the *DROSHA* amplifications observed in our study might be driven by the closely located *GOLPH3*, we reanalyzed this region with the use of the new 5p-arm-specific MLPA assay. This experiment confirmed *DROSHA* amplifications in analyzed samples and showed that amplification of *DROSHA* results mostly from the chromosome-level amplification of almost the entire 5p-arm. This experiment clearly demonstrated that amplification of *DROSHA* does not depend on the focal amplification of closely located *GOLPH3* or any other specific oncogene on the 5p-arm. Regardless of whether *DROSHA* and *DICER1* are drivers of their amplifications, the amplifications of these two key miRNA biogenesis genes may increase their expression and, as a consequence, may contribute to the global destabilization of miRNA expression observed in many types of cancer.

The computational analysis of publically available oncogenomic data showed that the copy number variation of *DROSHA* correlates well with its expression and that increased expression of *DROSHA* is associated with worse survival. The above analyses of oncogenomic data are in line with our experimental results suggesting decreased survival of patients with gain or amplification of *DROSHA* (Figure 5). A similar computational analysis of *DICER1* also showed a good correlation between its copy number categories and expression. However, in contrast to *DROSHA*, increased expression of *DICER1* was associated with longer survival in various cancers including lung cancer. Although such results must be interpreted with caution, the opposite effects of increased expression of *DROSHA* and *DICER1* on survival (positive and negative, respectively) may suggest the oncogenic role of intermediate products of these two enzymes, that is, pre-miRNAs (either specific or as a class). It should be noted that the advantage of the computational results discussed above is that they are based on independent (objectified) whole genome datasets generated in projects not focused specifically on *DICER1*, *DROSHA* or any other miRNA biogenesis gene.

Our results add to the complex picture of the role of *DICER1* and *DROSHA* in cancer. The miRNA biogenesis genes were primarily considered as haploinsufficient tumor suppressors [59]. This notion results mostly from the observation that the overall level of miRNAs is often reduced in cancer [60–62] and from the fact that germline loss-of-function mutations in *DICER1* are causative variants in the so called DICER1 syndrome, which is associated with increased risk of numerous, mostly early, childhood malignancies and benign tumors [63]. The representative (most common) malignancy for this syndrome is pleuropulmonary blastoma, which occurs in the lungs. More recently, analysis of cancers associated with DICER1 syndrome as well as other early childhood cancers (e.g., Wilms tumor) led to the identification of a peculiar pattern of somatic second-hit mutations in *DICER1* and *DROSHA*. These mostly missense mutations are not randomly distributed over the genes but form clear hotspots, mostly affecting few amino acid residues located in or adjacent to metal-ion-binding residues in the RNaseIIIb domain of either *DICER1* (D1709, E1813) or *DROSHA* (E1147, D1151) [63–68]. Functional analyses suggest that these mutations are not deleterious (as expected for typical second-hit mutations) but rather modify the function of DICER1 or DROSHA, making it favorable for cancer (oncogenic) (recently discussed in [69, 70]). It was shown that modified enzymes selectively reduce the processing of miRNAs generated from the 5′ arm of pre-miRNA hairpins and as a consequence modify the miRNA expression profile in cancer [65, 66, 71, 72].

Our results and the notion about the oncogenic role of *DROSHA* are very much in line with previous results suggesting that *DROSHA* is a key gene driving frequent gains of the 5p-arm in cervical squamous cell carcinoma (SCC) [73, 74]. Analysis of primary cervical SCC samples and cell lines showed that the frequent copy number gains and overexpression of *DROSHA* led to an altered profile of miRNA expression, including the expression of many cancer-related miRNAs. Among the miRNAs showing the highest overexpression was *miR-31*. Functional *in vitro* analyses (including wound healing test) showed that upregulation of *DROSHA* increases motility and invasiveness of squamous SCC cell lines [73, 74]. It was also shown that overexpression of *DROSHA* is associated with metastasis and decreased survival in esophageal cancer patients [75].

It should be noted that other genes of miRNA biogenesis enzymes may contribute to the regulation of global or individual miRNA expression in cancer. Therefore, to better understand and evaluate the impact of somatic copy number variation of miRNA biogenesis genes on miRNA expression in cancer, a more complex analysis is needed.

In conclusion, our results show a substantial somatic copy number variation in genomic regions comprising miRNA genes. Among these regions were those showing a substantial increase in the average copy number (frequently amplified), and regions with decreased average copy number. Concordance of copy number and expression changes of some miRNAs suggest that copy number variation may be an important mechanism responsible for regulation of these miRNAs in lung cancer. Therefore our observations support the proposed earlier notion, implying the high genomic instability of miRNA gene regions and contribution of copy number variation in the regulation of miRNA expression in cancer [29, 30]. It should be emphasized however that the amplitude and recurrence of copy number changes cannot be simply interpreted as the oncogenic role of a variable region/gene in cancer.

Our results also indicate the important role of miRNA biogenesis genes, especially *DROSHA*, in lung cancer. Even if these genes are not drivers of their copy number changes, they may affect global regulation of miRNA expression in cancer.

Finally, somatic copy number changes of some of the analyzed genes including *DROSHA* correlate with survival of cancer patients. Although the results of our survival analyses are only marginally significant (relatively low number of samples) and must be replicated in an independent group of samples, the copy number changes would be attractive biomarkers due to (i) the relatively high stability of genomic DNA, even extracted from formalin-fixed paraffin embedded (FFPE) samples; (ii) the small amount of DNA necessary for analysis; (iii) relatively low cost; (iv) simplicity; and (v) the reliability of copy number analysis. The drawback of such analysis is, however, contamination of the cancer samples with a difficult to estimate amount of normal DNA.

## MATERIALS AND METHODS

### Selection and processing of NSCLC samples for molecular analysis

We retrospectively reviewed a cohort of 254 patients with histopathologically confirmed NSCLC diagnosed at the Franciszek Lukaszczyk Oncology Center in Bydgoszcz (central Poland). The age of the patients ranged from 35 to 81. A total of 254 specimens that passed the quality control steps (microscopic analysis and tumor content qualification as well as qualitative and quantitative DNA analysis) were obtained following surgeries, fine-needle aspirations (FNAs), endobronchial ultrasound with guided transbronchial needle aspiration (EBUS-TBNA) procedures or pleural fluid sampling. The samples were stained with hematoxylin and eosin for the qualitative and quantitative analysis of tumor cells in the analyzed material (including macrodissection in marked out samples) as described previously [76]. The study was approved by the Committee of Ethics of Scientific Research of Collegium Medicum of Nicolaus Copernicus University, Poland (KB 265/2012). The data were analyzed anonymously.

DNA extraction was performed after the microdissection of a region indicated by the pathomorphologist, and the quality and quantity of DNA samples were evaluated as described previously [40].

### Copy number analysis by MLPA

MLPA analysis was performed with the use of three in-house designed and generated assays, LC-miR_1, LC-miR_2 and LC-5p. Both LC-miR_1 and LC-miR_2 assays contained 14 probes specific for 7 miRNA genes (two probes for each miRNA or miRNA-cluster gene), 3 probes specific for one of miRNA biogenesis gene, and 4 control probes (located on different chromosomes outside of chromosome 5 and regions of known cancer-related genes). The LC-5p assay contained 6 probes more or less evenly covering the short arm of chromosome 5 (5p-arm), 5 probes specific for *DROSHA*, 3 probes specific for *GOLPH3*, and 4 control probes. The detailed characteristics, genomic positions and sequences of all probes used in this study are presented in Supplementary Table S1.

The MLPA probes and the general layout of the probe sets were designed according to a previously proposed strategy [36, 37]. This strategy utilizes only short oligonucleotide probes that can easily be generated via standard chemical synthesis. Briefly, each probe was composed of two half-probes of equal size, and the total probe length ranged from 93 to 164 nt. The target sequences for the probes were selected to avoid SNPs, repeat elements and sequences of extremely high or low GC content. The MLPA probes were synthesized by IDT (Skokie, IL, USA).

The MLPA reactions were run according to the manufacturer's general recommendations (MRC-Holland, Amsterdam, the Netherlands), as described earlier in [37, 77]. All reagents except the probe mixes were purchased form MRC-Holland (http://www.mlpa.com). The products of the MLPA reaction were subsequently diluted 20x in HiDi formamide containing GS Liz600, which was used as a DNA sizing standard, and separated via capillary electrophoresis (POP7 polymer) in an ABI Prism 3130XL apparatus (Applied Biosystems, Carlsbad, CA, USA).

The obtained electropherograms were analyzed using GeneMarker software v2.4.0 (SoftGenetics, State College, PA, USA). The signal intensities (peak heights) were retrieved and transferred to prepared Excel sheets (available upon request). For each individual sample, the signal intensity of each probe was divided by the average signal intensity of the control probes to normalize the obtained values and to equalize run-to-run variation. Due to high signal variation, the control probe 3 (ctrl_3) was excluded from analysis. To calculate relative copy number value of particular probe, the normalized signal of this probe was divided by a corresponding value of this probe in the reference (non-cancer) sample and multiplied by 2. The relative copy number of a particular gene was calculated as an average of the normalized copy number value of 2 or 3 probes specific to this gene. If the difference between the maximum and minimum signal of the averaged probes was higher than one-third of an average copy number value or if the coefficient of variation of the averaged probes was higher than 0.3, the result was excluded from further analyses.

### Databases and statistical analysis

All statistical analyses were performed using Statistica (StatSoft, Tulsa, OK) or Prism v. 4.0 (GraphPad, San Diego, CA). All *p*-values were provided for two-sided tests. All human genome positions indicated in this report refer to the February 2009 (GRCh37/hg19) human reference sequence. The datasets for analysis and visualization of the relationship between copy number category and expression level of *DROSHA* and *DICER1* were obtained from cBioPortal for Cancer Genomics (MemorialSloan-Kettering Cancer Center, New York, NY, USA; http://www.cbioportal.org/) [42, 43] and were analyzed with the use of the cBioPortal Plots tool. The survival analyses of the cancer patients with high and low levels of either *DICER1* or *DROSHA* expression were performed with the use of datasets and tools available in the PPISURV portal (http://www.bioprofiling.de/GEO/PPISURV/ppisurv.html) [45].

## SUPPLEMENTARY FIGURES AND TABLES








