# Supplementary material for: High copy number variation of cancer-related microRNA genes and frequent amplification of DICER1 and DROSHA in lung cancer
Source: Oncotarget. 2015 Jun 22;6(27):23399–416. doi: 10.18632/oncotarget.4351 (PMC4695126; doi:10.18632/oncotarget.4351)
Supplement: Supplementary file 1 [file oncotarget-06-23399-s001.pdf]

SUPPLEMENTARY FIGURES AND TABLES

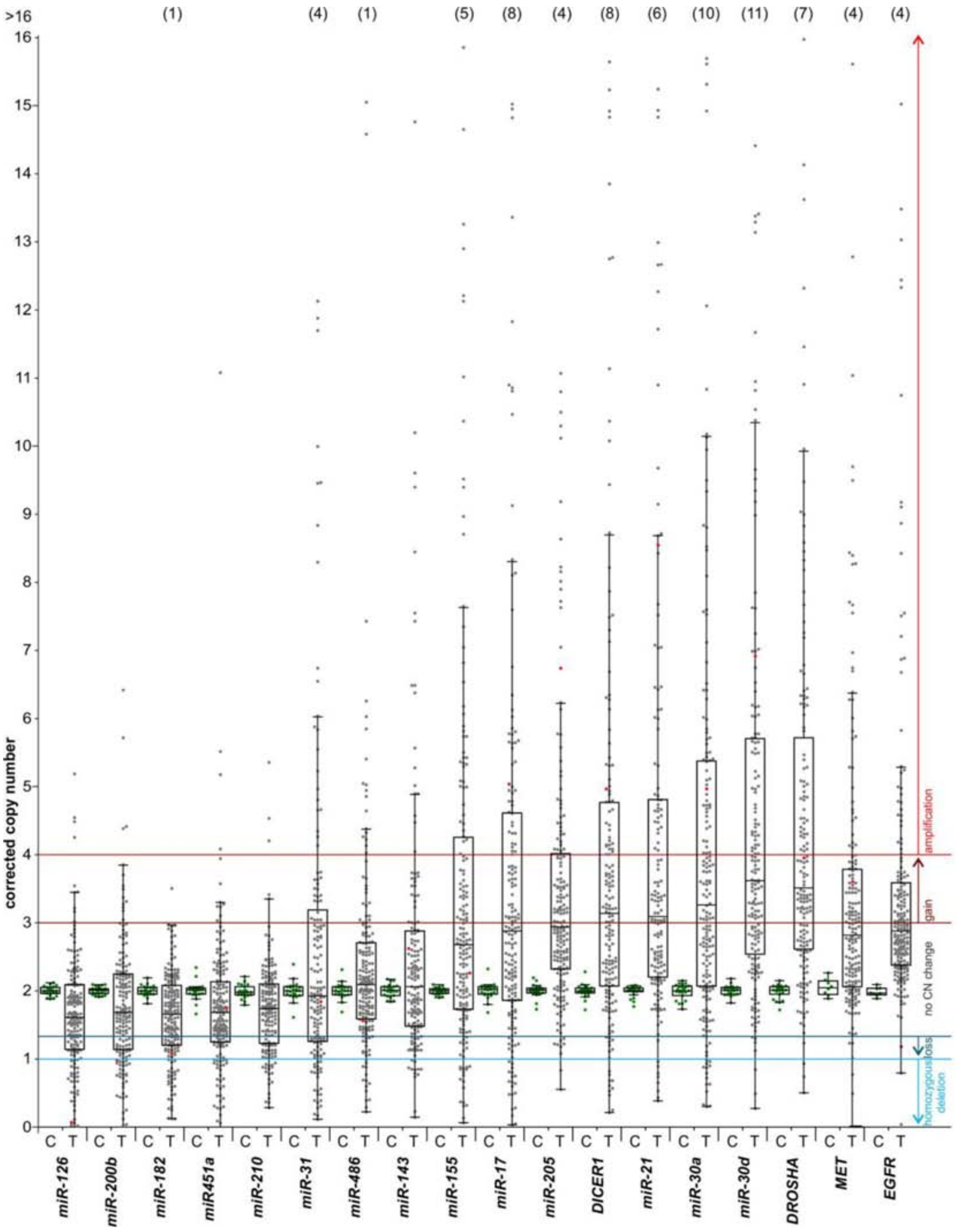

Supplementary Figure S1: Graphical summary of the copy number variation of the analyzed genes in NSCLC samples. The graph shows the results of copy number analysis of the selected miRNA and miRNA biogenesis genes as well as two lung cancer related oncogenes, *MET* and *EGFR*. The y-axis shows the copy number value corrected for PTC (dilution of cancer cells). Numbers in brackets (above graph) indicate samples with a copy number value >16. All other Figure legend details are the same as Figure 3A.

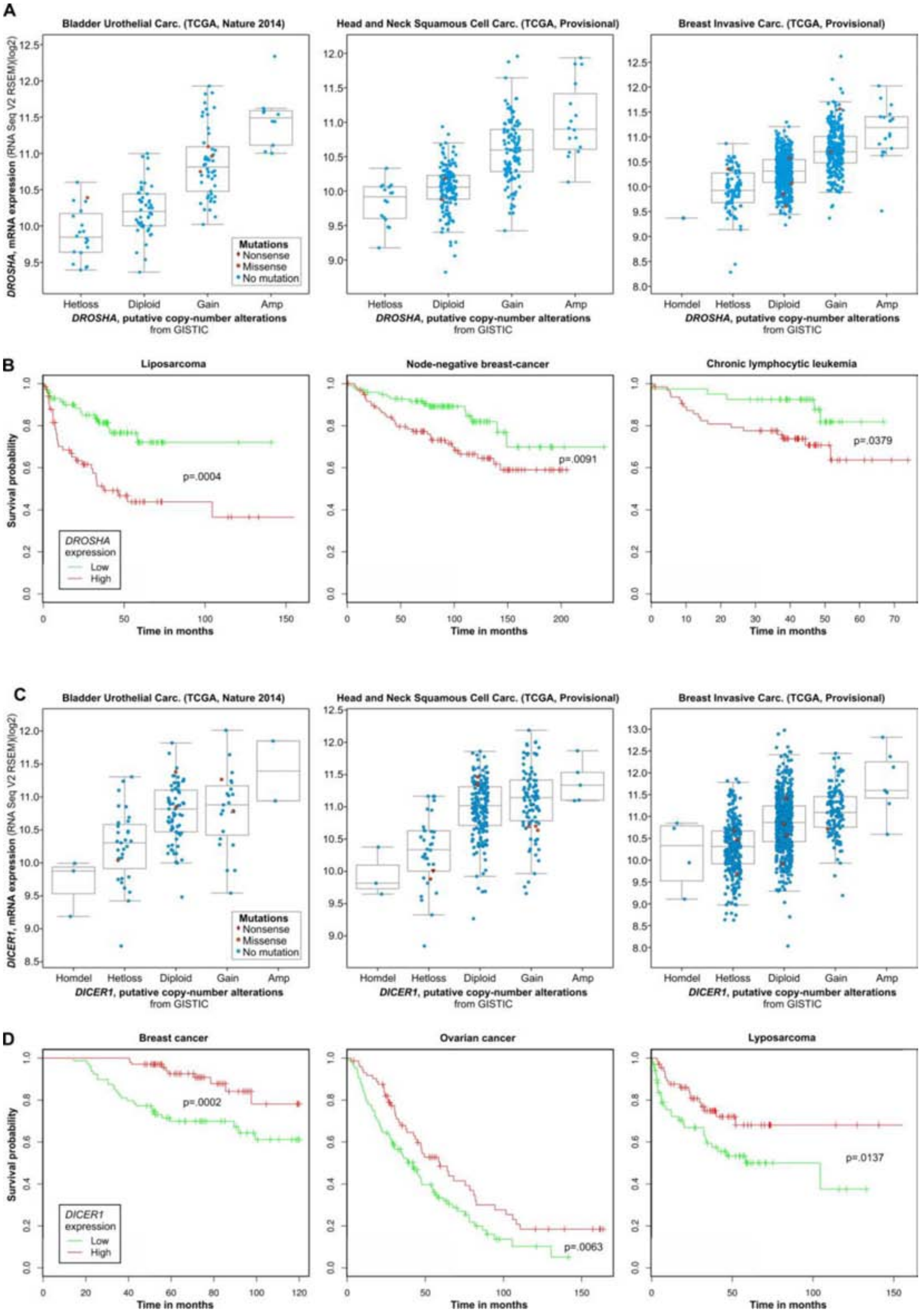

Supplementary Figure S2: Computational analysis of clinical (survival) and oncogenomic data of *DROSHA* and *DICER1* performed with the use of datasets representing different types of cancer. Mutual relation of copy number and expression (oncogenomic data) of *DROSHA* **A** and **B.** and *DICER1* **C** and **D.**, and the relation of their expression to survival of cancer patients (clinical data). A) and C) Correlation analysis of copy number categories and expression level, performed with the use of datasets (indicated above the graphs) deposited and tools available in cBioPortal for Cancer Genomics. B) and D) Survival analysis performed with the use of datasets (indicated above the graphs; GEO: GSE30929, GSE11121, GSE22762, GSE24450, GSE26712, GSE30929) deposited in and tools available from the PPISURV web-portal.

Supplementary Table S1. MLPA assays - detailed characteristics.

Supplementary Table S2. Relative copy number values of the analyzed regions.

Supplementary Table S3. Comparison of clinical data with copy number categories of analyzed miRNA and miRNA biogenesis genes.
