# Supplementary material for: High copy number variation of cancer-related microRNA genes and frequent amplification of DICER1 and DROSHA in lung cancer
Source: Oncotarget. 2015 Jun 22;6(27):23399–416. doi: 10.18632/oncotarget.4351 (PMC4695126; doi:10.18632/oncotarget.4351)
Supplement: Supplementary file 4 [file oncotarget-06-23399-s004.doc]

Supplementary Table S3. Comparison of clinical data with copy number categories of analyzed miRNA and miRNA biogenesis genes

| compared copy number categories | **age** | | | | **sex** | | | **tumor stage** | | | | | **remission** | | **progression** | | **metastasis** | |
| --- | --- | --- | --- | --- | --- | --- | --- | --- | --- | --- | --- | --- | --- | --- | --- | --- | --- | --- |
|  | <60/≥60 years | Fisher's exact test p-val | average (SD) | T test  p-val | male | female | Fisher's exact test p-val | I  number (%) | II  number (%) | III  number (%) | IV  number (%) | Mann-Whitney test p-val | number (%) | Fisher's exact test p-val | number (%) | Fisher's exact test p-val | number (%) | Fisher's exact test p-val |
| ***miR-126*** |  |  |  |  |  |  |  |  |  |  |  |  |  |  |  |  |  |  |
| del | 8/18 | 0.207 | 64.9(8.3) | 0.046 | 21 | 6 | 0.134 | 1(6) | 4(24) | 3(18) | 9(53) | 0.792 | 2(12) | 0.733 | 7(41) | 1 | 8(47) | 0.598 |
| others | 82/100 |  | 61.2(9) |  | 115 | 72 |  | 3(3) | 8(9) | 39(41) | 44(47) |  | 18(19) |  | 41(42) |  | 38(39) |  |
| ***miR-200b*** |  |  |  |  |  |  |  |  |  |  |  |  |  |  |  |  |  |  |
| del | 8/10 | 1.000 | 61.6(9) | 0.965 | 11 | 6 | 1.000 |  |  |  |  |  | 0(0) | 0.207 | 4(40) | 1 | 6(60) | 0.197 |
| others | 83/102 |  | 61.5(9.2) |  | 116 | 71 |  |  |  |  |  |  | 19(19) |  | 44(43) |  | 39(38) |  |
| ***miR-451a*** |  |  |  |  |  |  |  |  |  |  |  |  |  |  |  |  |  |  |
| del | 4/8 | 0.554 | 66.8(8.4) | 0.041 | 10 | 2 | 0.136 |  |  |  |  |  |  |  |  |  |  |  |
| others | 89/107 |  | 61.2(9.2) |  | 119 | 78 |  |  |  |  |  |  |  |  |  |  |  |  |
| ***miR-31*** |  |  |  |  |  |  |  |  |  |  |  |  |  |  |  |  |  |  |
| del | 7/17 | 0.126 | 65.7(7.7) | 0.017 | 13 | 11 | 0.375 | 0(0) | 1(10) | 6(60) | 3(30) | 0.432 |  |  |  |  |  |  |
| others | 83/93 |  | 61(9.1) |  | 113 | 64 |  | 3(3) | 10(10) | 37(38) | 47(48) |  |  |  |  |  |  |  |
| ***miR-31*** |  |  |  |  |  |  |  |  |  |  |  |  |  |  |  |  |  |  |
| amp+gain | 12/12 | 0.665 | 61(10.9) | 0.761 | 15 | 10 | 0.659 | 0(0) | 3(17) | 7(39) | 8(44) | 0.767 | 6(33) | 0.093 | 4(22) | 0.074 | 8(44) | 0.794 |
| others | 78/98 |  | 61.6(8.8) |  | 121 | 65 |  | 3(3) | 8(9) | 36(4) | 42(47) |  | 14(15) |  | 42(46) |  | 36(39) |  |
| ***miR-143*** |  |  |  |  |  |  |  |  |  |  |  |  |  |  |  |  |  |  |
| amp+gain | 6/5 | 0.758 | 61.6(12.5) | 0.972 | 6 | 5 | 0.756 |  |  |  |  |  | 4(36) | 0.215 | 2(18) | 0.108 | 5(45) | 0.532 |
| others | 79/90 |  | 61.7(8.9) |  | 103 | 67 |  |  |  |  |  |  | 16(17) |  | 44(47) |  | 34(36) |  |
| ***miR-155*** |  |  |  |  |  |  |  |  |  |  |  |  |  |  |  |  |  |  |
| amp+gain | 19/27 | 1.000 | 62.8(10.3) | 0.394 | 25 | 21 | 0.235 | 0(0) | 4(14) | 11(39) | 13(46) | 0.956 | 7(24) | 0.795 | 11(38) | 0.669 | 11(38) | 0.83 |
| others | 71/95 |  | 61.5(8.8) |  | 107 | 60 |  | 4(5) | 8(10) | 33(39) | 39(46) |  | 14(16) |  | 37(43) |  | 35(41) |  |
| ***miR-17*** |  |  |  |  |  |  |  |  |  |  |  |  |  |  |  |  |  |  |
| amp+gain | 24/32 | 0.875 | 61.9(9.8) | 0.736 | 34 | 22 | 0.872 | 1(3) | 3(9) | 12(35) | 18(53) | 0.363 | 7(20) | 0.795 | 15(43) | 1 | 13(37) | 0.837 |
| others | 69/84 |  | 61.4(9) |  | 97 | 57 |  | 3(4) | 9(12) | 32(41) | 34(44) |  | 14(18) |  | 34(43) |  | 32(4) |  |
| ***miR-205*** |  |  |  |  |  |  |  |  |  |  |  |  |  |  |  |  |  |  |
| amp+gain | 20/26 | 0.867 | 61.9(11) | 0.696 | 28 | 18 | 0.865 | 2(6) | 5(16) | 8(25) | 17(53) | 0.804 | 8(24) | 0.302 | 12(36) | 0.409 | 13(39) | 1 |
| others | 75/88 |  | 61.3(8.6) |  | 102 | 62 |  | 2(3) | 7(9) | 36(46) | 34(43) |  | 13(16) |  | 37(46) |  | 31(38) |  |
| ***DICER1*** |  |  |  |  |  |  |  |  |  |  |  |  |  |  |  |  |  |  |
| amp+gain | 31/32 | 0.359 | 60.9(10) | 0.433 | 38 | 25 | 0.641 | 2(5) | 5(13) | 14(35) | 19(48) | 0.715 | 8(20) | 0.800 | 17(43) | 1 | 15(38) | 0.84 |
| others | 57/80 |  | 62(8.8) |  | 88 | 50 |  | 2(3) | 4(6) | 29(44) | 31(47) |  | 12(17) |  | 29(42) |  | 28(41) |  |
| ***miR-21*** |  |  |  |  |  |  |  |  |  |  |  |  |  |  |  |  |  |  |
| amp+gain | 31/34 | 0.641 | 60.7(9.9) | 0.254 | 37 | 29 | 0.344 | 2(4) | 7(13) | 19(37) | 24(46) | 0.785 | 11(20) | 1.000 | 20(37) | 0.331 | 23(43) | 0.428 |
| others | 50/66 |  | 62.3(8.5) |  | 74 | 42 |  | 2(4) | 4(8) | 22(42) | 24(46) |  | 10(19) |  | 25(47) |  | 18(34) |  |
| ***miR-30a*** |  |  |  |  |  |  |  |  |  |  |  |  |  |  |  |  |  |  |
| amp+gain | 36/52 | 0.674 | 61.9(9.6) | 0.814 | 53 | 35 | 0.568 | 2(4) | 8(16) | 17(34) | 23(46) | 0.849 | 11(22) | 0.466 | 18(36) | 0.255 | 21(42) | 0.7 |
| others | 56/69 |  | 61.6(8.8) |  | 81 | 45 |  | 2(3) | 4(6) | 27(44) | 29(47) |  | 10(15) |  | 31(48) |  | 24(37) |  |
| ***miR-30d*** |  |  |  |  |  |  |  |  |  |  |  |  |  |  |  |  |  |  |
| amp+gain | 35/50 | 0.569 | 61.5(9.4) | 1.000 | 53 | 33 | 1.000 | 1(2) | 6(12) | 21(41) | 23(45) | 0.917 | 10(19) | 1.000 | 22(42) | 1 | 21(40) | 1 |
| others | 53/64 |  | 61.5(8.7) |  | 72 | 45 |  | 3(5) | 6(11) | 22(39) | 26(46) |  | 11(19) |  | 24(41) |  | 24(41) |  |
| ***DROSHA*** |  |  |  |  |  |  |  |  |  |  |  |  |  |  |  |  |  |  |
| amp+gain | 33/57 | 0.144 | 60.7(10.1) | 0.286 | 46 | 35 | 0.175 | 2(4) | 5(10) | 17(35) | 24(50) | 0.552 | 10(20) | 0.807 | 19(37) | 0.340 | 22(43) | 0.556 |
| others | 49/53 |  | 62.1(8) |  | 75 | 37 |  | 2(3) | 7(12) | 24(41) | 25(43) |  | 21(36) |  | 27(47) |  | 10(17) |  |

Somewhat different total numbers of samples in copy number categories of particular miRNAs results form different number of samples with undetermined copy number. The statistical analysis was not performed if number of samples in one of compared copy number categories was <10. SD – standard deviation,
